# Comparing Lean and Obese PCOS in Different PCOS Phenotypes: Evidence That the Body Weight Is More Important than the Rotterdam Phenotype in Influencing the Metabolic Status

**DOI:** 10.3390/diagnostics12102313

**Published:** 2022-09-25

**Authors:** Enrico Carmina, Rogerio A. Lobo

**Affiliations:** 1 Endocrinology Unit, University of Palermo School of Medicine, 90133 Palermo, Italy; 2 Department of Obstetrics and Gynecology, Columbia University, New York, NY 10027, USA

**Keywords:** PCOS, lean PCOS, obese PCOS, PCOS phenotypes, insulin resistance in PCOS

## Abstract

Polycystic Ovary Syndrome (PCOS) represents a heterogeneous disorder and, using Rotterdam diagnostic criteria, four main phenotypes (A, B, C, and D) have been distinguished. However, it remains unclear whether lean versus obesity status influences findings in the various phenotypes of women with PCOS. 274 women with PCOS were consecutively assessed. Among these women, there were 149 with phenotype A, 24 with phenotype B, 94 with phenotype C, and 7 with phenotype D. We found normal body weight to be very common (65%) in phenotype C patients, common (43%) in phenotype A and D patients, and less represented (but still 25%) in phenotype B patients. Obesity was common in phenotype B (54%) and phenotype A (33%) patients and uncommon in phenotype C (only 11%) and phenotype D (14%) patients. Obese and lean patients of each phenotype were compared. Compared to the phenotype C PCOS patients, both phenotype A and B patients had higher total testosterone circulating values and higher luteinizing hormone/follicle stimulating hormone (LH/FSH) ratio (*p* < 0.01) while anti-Mullerian hormone (AMH) levels were higher only in phenotype A PCOS patients. Instead, in the three obese PCOS phenotypes no differences in serum insulin, Homeostatic Model Assessment of Insulin Resistance (HOMA-IR) calculation, and lipid blood values were observed. Analysis of data of lean patients gave similar results. Compared to the phenotype C PCOS patients, both phenotype A and B patients had higher total testosterone circulating values and higher LH/FSH ratio (*p* < 0.01) while AMH levels were higher only in phenotype A PCOS patients. However, no differences were observed in the circulating insulin levels, HOMA-IR calculation, or blood lipids between the three groups of lean PCOS patients. We conclude that Rotterdam phenotypes express the differences between PCOS patients in terms of ovulatory pattern and androgen secretion but fail to differentiate between obese patients with altered metabolic patterns and lean patients with normal metabolic patterns. A new classification of PCOS patients is needed and it should consider the influence of body weight on the metabolic patterns of PCOS patients.

## 1. Introduction

Polycystic Ovary Syndrome (PCOS) represents a common but heterogeneous disorder and, using Rotterdam diagnostic criteria, four main phenotypes (A, B, C, and D) have been distinguished [1,2,3,4,5]. However, this classification only partially expresses the large variability of PCOS. It has remained unclear whether lean PCOS patients represent a different PCOS phenotype.

In the past 20 years, little attention has been given to this issue probably because in the USA the large majority of patients with PCOS are obese or overweight [6,7,8,9] and studies on Rotterdam PCOS phenotypes have reported important differences in mean body mass index (BMI) between phenotypes [4,10,11]. Women with PCOS who have phenotypes A and B, meaning they are anovulatory, are generally obese and are considered to be affected by a more severe form of PCOS with consequences not only for fertility but also for metabolic processes [4,10,11]. On the contrary, women with phenotype C who have regular ovulatory menses are generally lean or slightly overweight with no or little metabolic problems [4,10,11]. In addition, our previous data have shown that some patients may change their phenotypic status over time, from phenotype A or B to phenotype C (ovulatory) by reducing body weight [12].

However, in many countries (mainly in the Mediterranean area) a large proportion of women with phenotype A or B have been found to have normal body weight from the onset of the diagnosis of the disorder, which is independent of diet [11,13,14,15,16,17].

In this study, we aimed to determine if PCOS patients with Rotterdam phenotypes should be further divided into different PCOS sub-phenotypes according to their body weight. It may have important consequences in terms of laboratory evaluation, follow up appointments and therapeutic approaches.

## 2. Material and Methods

Between November 2018 and April 2022, 274 consecutive patients with a diagnosis of PCOS were studied. These patients were referred because of hyperandrogenism and/or menstrual disorders. None of these patients were included in our previous studies regarding PCOS phenotypes [2,10,11].

The diagnosis of PCOS was based on Rotterdam criteria, two out of three of the following criteria: chronic anovulation, clinical or biologic hyperandrogenism, and/or polycystic ovaries on ultrasound, after the exclusion of other medical disorders [1].

Phenotype A PCOS was diagnosed in patients presenting with all three characteristics of the syndrome (chronic anovulation, hyperandrogenism and polycystic ovaries). Phenotype B PCOS was diagnosed in patients presenting with hyperandrogenism and chronic anovulation but no polycystic ovaries, Phenotype C PCOS was diagnosed in patients presenting with hyperandrogenism and polycystic ovaries in the presence of ovulatory cycles and Phenotype D PCOS was diagnosed in women presenting with chronic anovulation and polycystic ovaries but normal androgen status (no hirsutism or increased circulating androgens) [1,2,3,4,5].

In all patients, serum levels of luteinizing hormone (LH), follicle stimulating hormone (FSH), Estradiol, total testosterone (T), dehydroepiandrosterone sulfate (DHEAS), 17-hydroxy-progesterone (17OHP) and anti-Mullerian hormone (AMH) were determined on days 3–5 of the cycle. In non-menstruating women blood samples were obtained after withdrawal bleeding after progestogen administration. Normally menstruating patients had serum progesterone measured on days 21–22 of the cycle.

Anovulation was defined as serum progesterone <3 ng/mL (<9.54 nmol/L). In patients with normal menses, at least two consecutive menstrual cycles were studied and a finding of low levels of serum progesterone (<3 ng/mL) in both cycles indicated the presence of chronic anovulation.

Clinical hyperandrogenism was defined as the presence of hirsutism. Hirsutism was assessed by Ferriman-Gallwey-Lorenzo scores [18], and patients with scores higher than 6 were considered hirsute. Adult acne and female pattern hair loss were not considered a sign of hyperandrogenism if androgen levels were normal [19,20].

Biochemical hyperandrogenism was defined as serum testosterone > 34 ng/dL and/or serum DHEAS higher than 3 mcg/mL (>7.8 mmol/L). Total testosterone was determined by mass spectrometry after liquid chromatography (LC/MS) assay while the other steroid hormones were measured by specific RIAs using previously described methods [21]. In all patients, serum 17OH progesterone values were determined to exclude the existence of Non-Classic Congenital Adrenal Hyperplasia [22]. In some patients, because of clinical suspicion, urinary free cortisol, and serum prolactin and TSH were measured by commercial RIA methods to exclude other endocrine conditions.

For AMH measurement, samples were collected into serum tubes with gel separators and centrifuged within 5 h. AMH was measured using a previously described method [23]. The conversion of AMH in ng/mL to pmol/L requires that values be multiplied by 7.143.

LH and FSH were measured by specific RIAs using previously described methods [21].

In all assays, intra-assay and interassay coefficients of variation did not exceed 6% and 15%, respectively.

Transvaginal pelvic ultrasound was performed using a transducer frequency of 8–10 MHz and the presence of polycystic ovaries was established by the finding of increased number of follicles, each of which measured 2–10 mm in diameter and/or increased ovarian size [23,24].

In all women with PCOS a metabolic profile was obtained. The metabolic profile included measurements of fasting blood levels of glucose, insulin, total cholesterol, high density lipoprotein-cholesterol (HDL-C), low density lipoprotein-cholesterol (LDL-C) and triglycerides (TG). Insulin sensitivity was evaluated by the quantitative HOMA-IR method [25].

No patient had received any medication for at least 3 months before the study, and all patients gave informed consent for this evaluation and the research protocol had obtained institutional approval from the ethical committee of our university (2018/23).

The various values of the women with PCOS were compared to those of sixty-five normal ovulatory women. These controls were drawn from the same population and did not report complaints of hyperandrogenism or menstrual irregularities.

## 3. Statistical Analysis

Statistical analyses were performed using Statview 5.0 (SAS Institute, Cary, NC, USA). Due to several values not being normally distributed, a log transformation was necessary to obtain a normal distribution. Mann-Whitney U tests were performed to compare parameters between the PCOS and control groups. Analysis of variance (ANOVA) followed by Tukey tests was performed to assess differences in clinical and biochemical parameters between different phenotypes. The accuracy of parameters used to discriminate between the various phenotypes of PCOS and controls was evaluated using ROC curve analyses. Differences in reliability between different parameter values were assessed by Tukey multiple comparison tests. *p* < 0.05 was considered statistically significant. All results are reported as mean ± SD.

## 4. Results

Compared to controls, the cohort of women with PCOS had similar age (mean age 24.2 ± 5 versus 24 ± 3 years) but a higher (*p* < 0.01) mean body weight (mean BMI 26.7 ± 6 versus 23.6 ± 4). PCOS women had a higher (*p* < 0.01) prevalence of obesity (27% versus 6%) compared to controls.

Phenotype A PCOS was diagnosed in 149 women (54%), phenotype B PCOS in 24 women (9%), phenotype C PCOS in 94 women (34%), and phenotype D PCOS in 7 women (3%).

Characteristics of the 4 phenotypes of women with PCOS were compared (Table 1).

Mean age, blood estradiol, glucose, total and HDL cholesterol were similar in the different PCOS phenotypes while blood values of AMH were significantly (*p* < 0.01) increased only in phenotype A.

Phenotype A and B PCOS patients had significantly (*p* < 0.01) higher BMI and values of LH/FSH ratio, total testosterone, insulin, HOMA-IR, and triglycerides than phenotype C PCOS women. Compared to other phenotypes, patients with phenotype D had significantly (*p* < 0.01) lower levels of T while BMI and LH/FSH ratio values were like those found in phenotype C.

In Table 2, the prevalence of lean, overweight, and obese patients in the different PCOS phenotypes are reported. Obesity was most common in phenotype B PCOS patients (54%) but was common also in phenotype A PCOS women (43%) and uncommon in phenotype C (11%) and phenotype D (14%) PCOS patients. Normal body weight was very common (65%) in phenotype C, still common in phenotype A (43%) and phenotype D (43%), and less represented (25%) in phenotype B PCOS patients. Overweight patients were about a fourth of all patients in the three main phenotypes, without large differences between them, and slightly more (43%) in phenotype D patients.

Patients were divided into three subgroups according to their body weight (obese, overweight, and normal weight). Due to the small number of patients, this analysis did not include patients with phenotype D.

Obese and lean patients of each phenotype were compared. Compared to lean patients of the same phenotype, obese patients of each main phenotype (A, B, or C) had significantly (*p* < 0.01) higher insulin, HOMA-IR, and Triglycerides and significantly (*p* < 0.01) lower AMH and HDL.C circulating levels. Obese patients of Phenotype A and B had also significantly (*p* < 0.01) higher T levels and lower LH/FSH ratios than lean patients of the same phenotype. No significant difference in these parameters between obese and lean patients with Phenotype C was found.

Obese patients of all 3 main phenotypes were compared (Table 3). Compared to the phenotype C PCOS patients, both phenotype A and B patients had higher total testosterone circulating values and higher LH/FSH ratio (*p* < 0.01) while AMH levels were higher only in phenotype A PCOS patients. Instead, in the three obese PCOS phenotypes no differences in serum insulin, HOMA-IR calculation, and lipid blood values were observed (Table 3).

Analysis of data of lean patients gave similar results (Table 4). Compared to the phenotype C PCOS patients, both phenotype A and B patients had higher total testosterone circulating values and higher LH/FSH ratio (*p* < 0.01) while AMH levels were higher only in phenotype A PCOS patients. However, no differences in insulin circulating levels, HOMA-IR calculation, or blood lipids between the three groups of lean PCOS patients were observed (Table 4).

## 5. Discussion

Initially, after the development of hormone measurements, PCOS was diagnosed in patients with increased LH or LH/FSH ratio [26]. However, these simple hormonal criteria exclude many patients with the disorder. After the NIH consensus conference, the diagnosis of PCOS was enlarged to include all women presenting irregular menses (chronic anovulation) and clinical or biologic hyperandrogenism [27]. Again, these diagnostic criteria excluded many patients previously diagnosed with PCOS. In the United Kingdom and other parts of Europe, NIH criteria were never used and the diagnosis was based on ultrasound morphology (and hyperandrogenism) [28].

However, it became progressively evident that the syndrome is very heterogeneous and may also include women with normal, ovulatory menses [29]. In fact, we reported that many ovulatory women present all characteristics of PCOS and mainly the two main pathogenetic elements: hyperandrogenism and Insulin resistance, and concluded that there was no reason to make the diagnosis of PCOS only in patients with chronic anovulation [30].

Finally, the introduction of Rotterdam diagnostic criteria (chronic anovulation, hyperandrogenism, and polycystic ovaries, two out of three) has partially expressed the possible clinical and hormonal characteristics of patients affected by PCOS. In fact, patients with PCOS may be anovulatory and hyperandrogenic with polycystic ovaries (phenotype A), but also may have normal ovaries (phenotype B) or be ovulatory (phenotype C) or also have normal circulating androgens and no clinical signs of androgen excess (phenotype D) [1,2,3,4,5].

However, this distinction in 4 phenotypes does not consider another important difference between PCOS patients. PCOS patients may be obese with severe insulin resistance and altered lipid patterns but may be also lean with mild or no clinically evident insulin resistance and with normal lipid patterns. While obesity is more common in PCOS than in the general population [2,3,4,5,6], many patients with all characteristics of the syndrome have normal body weight [4,5,6]. The mechanisms determining the increased prevalence of obesity in PCOS have never been clarified [4] but may be linked in some way to more severe insulin resistance [3,4,5].

Nevertheless, because patients with Phenotype A and B compared to Phenotype C and Phenotype D have higher mean body weight, insulin levels, and insulin resistance and more altered lipid pattern [10,11], it has been suggested that the Rotterdam classification may indeed include differences in insulin resistance and lipid pattern due to the majority of patients with Phenotype A or B being obese with metabolic alterations and more severe insulin resistance while most patients with Phenotype C lean with mild insulin resistance and no altered lipid profile [3,4,5,11].

Our study shows that, at least in a Mediterranean population but also in many other populations [11,12,13,14,15,16,17,31,32,33,34,35], this assumption is false. In our large population of 274 consecutive women with PCOS studied in a short period of time, most patients with Phenotype A were lean with no differences in terms of insulin resistance and lipid pattern with lean Phenotype C PCOS patients. Only in the less common Phenotype B, were the obese patients the majority although a fourth of the patients presenting with this phenotype were lean with a similarly normal pattern of insulin resistance and lipid profile.

Obesity was uncommon (only 10%) in Phenotype C patients but, interestingly, also the obese patients with this ovulatory phenotype had increased insulin resistance and altered lipid profile similar to that found in obese Phenotype A and B PCOS patients.

In some countries, like the USA, the prevalence of obesity in women with PCOS (and in the general population) is much higher (60–70%) [6,9]. However, in these countries, at least one-third of the PCOS patients diagnosed by Rotterdam criteria are lean or slightly overweight [8,9].

We conclude that Rotterdam phenotypes well express differences between PCOS patients in terms of ovulatory pattern and androgen secretion but fail to differentiate between obese patients with altered metabolic patterns and lean patients with normal metabolic patterns. In fact, independently on their A, B or C phenotype, metabolic alterations were related to body weight with obese patients of the three phenotypes presenting elevated insulin levels, increased insulin resistance, and altered lipid profiles. Lean PCOS patients, also inside the A and B phenotypes, had normal insulin circulating levels, no clinically relevant insulin resistance, and normal lipid profile.

The difference in metabolic patterns between lean and obese PCOS patients is particularly important in the classic (NIH) phenotypes A and B. In fact, in these phenotypes, obesity is common and is linked to severe metabolic alterations, but normal weight is also common and is associated with a completely different (and clinically normal) metabolic pattern. These data also suggest that mechanisms determining chronic anovulation in PCOS are not linked, or not linked exclusively, to metabolic alterations like insulin resistance.

More studies are needed but a new classification of PCOS patients considering the body weight of PCOS patients, independently of their ovulatory status, is necessary. It will improve our ability to make a correct diagnosis and to determine the best treatment and follow-up for these patients. It may be also be useful for a better understanding of the pathogenesis of the syndrome.

## Figures and Tables

**Table 1:** Some clinical and hormonal values of 149 Phenotype A, 24 Phenotype B, 94 Phenotype C and 7 Phenotype D patients with Polycystic Ovary Syndrome (PCOS).

	BMI	AMH ng/mL	LH/FSH Ratio	Total T ng/mL	Insulin mU/mL	HOMA-IR	HDL-C mg/dL	TG mg/dL
**Phenotype** **A** **PCOS**	28 ± * 6	10.2 ± ** 5	1.8 ± * 0.9	52 ± * 19	16 ± °° 8	3.3 ± °° 1.9	55 ± 11	90 ± °° 43
**Phenotype** **B** **PCOS**	30 ± * 8	5.2 ± 2	1.8 ± * 0.8	51 ± * 18	16 ± * 7	3.3 ± * 1.6	52 ± 12	99 ± * 67
**Phenotype** **C** **PCOS**	24.3 ± 4.6	6 ± 4	1.1 ± 0.5	42 ± ° 16	12 ± 7	2.5 ± 1.2	55 ± 12	78 ± 35
**Phenotype** **D** **PCOS**	25.2 ± 6	5.3 ± 2.8	1.3 ± 0.5	28 ± 6	15 ± 10	3 ± 1.8	55 ± 15	86 ± 58

BMI: Body mass index; AMH: Anti Mullerian Hormone; LH/FSH ratio: luteinizing hormone/follicle stimulating hormone; T: Testosterone; HOME-IR: Homeostatic Model Assessment of Insulin Resistance; HDL-C: High density lipoprotein Cholesterol; TG: Triglycerides; * *p* < 0.01 versus Phenotype C and D PCOS patients; ** *p* < 0.01 versus Phenotype B, C and D PCOS patients; ° *p* < 0.01 versus Phenotype D patients; °° *p* < 0.01 versus Phenotype C patients.

**Table 2:** Prevalence of different body weights for patients in different PCOS phenotype groups. Lean: BMI 18.5–24.9, overweight: BMI 25–29.9, obese: BMI ≥ 30.

	Phenotype A	Phenotype B	Phenotype C	Phenotype D
**Lean patients** **[*n* (%)]**	64 (43%)	6 (25%)	61 (65%)	3 (43%)
**Overweight** **[*n* (%)]**	36 (24%)	5 (21%)	23 (24%)	3 (43%)
**Obese** **[*n* (%)]**	49 (33%)	13 (54%)	10 (11%)	1 (14%)
**Total** **[*n* (%)]**	149 (100%)	24 (100%)	94 (100%)	7 (100%)

**Table 3:** Some hormonal and metabolic blood values in obese patients with different PCOS phenotypes.

	BMI	AMH ng/mL	LH/FSH Ratio	Total T (ng/dL)	Insulin mU/mL	HOMA-IR	HDL-C mg/dL	TG mg/dL
**Phenotype** **A** **PCOS**	35 ± 4	9.7 ± * 4	1.5 ± * 0.7	58 ± * 20	22 ± * 10	4 ± 2.2	48 ± 10	109 ± 55
**Phenotype** **B** **PCOS**	36 ± 6	5.1 ± 3	1.7 ± * 0.9	56 ± * 19	20 ± 6	3.9 ± 1.7	46 ± 11	112 ± 65
**Phenotype** **C** **PCOS**	34 ± 3	5.7 ± 3	1 ± 0.4	46 ± 11	18 ± 6	3.7 ± 1.5	48 ± 12	106 ± 65

AMH: anti-Mullerian hormone, LH/FSL: luteinizing hormone/follicle stimulating hormone, T: testosterone, HOMA-IR: Homeostatic Model Assessment of Insulin Resistance, HDL-C: high-density lipoprotein Cholesterol, TG: triglycerides. * *p* < 0.01 versus phenotype C PCOS patients.

**Table 4:** Some hormonal and metabolic blood values in lean patients with different PCOS phenotypes.

	BMI	AMH ng/mL	LH/FSH Ratio	Total T (ng/dL)	Insulin mU/mL	HOMA-IR	HDL-C mg/dL	TG mg/dL
**Phenotype** **A** **PCOS**	22 ± 2	10.7 ± * 6	2 ± * 1	50 ± * 18	10.6 ± 4	2.2 ± 1	56 ± 13	76 ± 28
**Phenotype** **B** **PCOS**	21 ± 2	6.1 ± 2	2 ± * 1	51 ± * 19	10 ± 4	2.2 ± 0.9	53 ± 15	71 ± 15
**Phenotype** **C** **PCOS**	21.5 ± 2	6.4 ± 4	1.1 ± 0.5	42 ± 19	10.4 ± 6	2.1 ± 1.2	57 ± 12	69 ± 27

AMH: anti-Mullerian hormone, LH/FSL: luteinizing hormone/follicle stimulating hormone, T: testosterone, HOMA-IR: Homeostatic Model Assessment of Insulin Resistance, HDL-C: high-density lipoprotein Cholesterol, TG: triglycerides. * *p* < 0.01 versus phenotype C PCOS.

## Data Availability

Data supporting results can be found at the office of Prof Carmina.
